# The brain structural and cognitive basis of odor identification deficits in mild cognitive impairment and Alzheimer’s disease

**DOI:** 10.1186/s12883-014-0168-1

**Published:** 2014-08-26

**Authors:** Grete Kjelvik, Ingvild Saltvedt, Linda R White, Pål Stenumgård, Olav Sletvold, Knut Engedal, Kristina Skåtun, Ann Kristin Lyngvær, Hill Aina Steffenach, Asta K Håberg

**Affiliations:** 1 Department of Circulation and Medical Imaging, Norwegian University of Science and Technology (NTNU), Trondheim, Norway; 2 Department of Geriatrics, St. Olav’s Hospital, University Hospital of Trondheim, Trondheim, Norway; 3 Department of Neuroscience, Medical Faculty, Pb. 8905, Norwegian University of Science and Technology (NTNU), Trondheim, 7491, Norway; 4 Department of Neurology, St. Olav’s Hospital, University Hospital of Trondheim, Trondheim, Norway; 5 Norwegian Centre for Ageing and Health, Department of Geriatric Medicine, Oslo University Hospital, Oslo, Norway; 6 Division of Mental Health and Addiction, Oslo University Hospital, Oslo, Norway; 7 Department of Medical Imaging, St. Olav’s Hospital, University Hospital of Trondheim, Trondheim, Norway

**Keywords:** Dementia, B-SIT, Hippocampus, Neurodegeneration, Olfaction, SSIT

## Abstract

**Background:**

The objectives of this study were to explore the relationship between olfactory impairment, cognitive measures, and brain structure volumes in healthy elderly individuals, compared to patients with amnestic mild cognitive impairment (aMCI) or early Alzheimer’s disease (AD). The primary aim was to elucidate possible differences in cognitive scores and brain structure volumes between aMCI/AD patients with relatively intact odor identification (OI) ability and those with reduced ability.

**Methods:**

Twelve patients with aMCI, six with early AD, and 30 control subjects were included. OI abilities were assessed with the Brief Smell Identification Test (B-SIT) and Sniffin Sticks Identification Test (SSIT). Neuropsychological tests of executive functions and memory were performed. Brain structural volumes were obtained from T1 weighted 3D MRI at 3 Tesla. Statistical comparisons between the patients with aMCI and AD indicated no significant differences in performance on most tests. Since the groups were small and AD patients were in an early phase of disease, all patients were subsequently considered together as a single group for studying OI. Patients were subdivided into relatively intact (scores >50%) and reduced OI (≤ 50% score) on the olfactory tests.

**Results:**

The aMCI/AD group with reduced OI ability, as measured by both B-SIT and SSIT, had significantly smaller hippocampal volume as compared to the patient group with OI scores > 50%. There was a significant association between OI scores and hippocampal volume in the patient (not the control) group. Similar changes with tests of executive function and memory were not found. Low OI scores on B-SIT were associated with conversion from aMCI to AD in patients. The reduced OI patient group was significantly faster on Rey complex figure copying than the fairly intact OI group.

**Conclusion:**

The results from this pilot study suggest that the reduction in the size of hippocampus in connection with early AD is associated more with loss of OI ability rather than loss of memory, thus demonstrating that impaired OI is an early marker of medial temporal lobe degeneration.

## Background

Tests of odor identification (OI) can discriminate neurodegenerative diseases, such as Alzheimer’s disease (AD) from normal aging, with high sensitivity and specificity [1]. The first observed olfactory deficit in patients with AD is not the ability to detect various odors, but to identify them correctly [2],[3].

Olfactory deficits have also frequently been reported in mild cognitive impairment (MCI) [4],[5], and there seem to be no significant differences between non-amnestic and amnestic MCI (aMCI) regarding OI performance [6]. Interestingly, studies using the Brief Smell Identification Test (B-SIT) show that both probable AD patients [7] and MCI subtypes score throughout the range of the test [6], thereby indicating that some probable AD and MCI patients have a relatively intact OI ability. OI ability can therefore not be used to distinguish patients with MCI from those with AD [8]. The reason why some patients retain OI ability longer than others is unknown, and we wished to investigate this further in the present study.

Pathological changes in different areas of the olfactory system have been suggested to cause the deficits of OI function in patients with AD, as well as other forms of dementia [9]. The OI deficit has been shown to correlate with the number of tangles in the entorhinal cortex and hippocampus in AD pathology [10]. Neuroimaging studies support a particular role for the medial temporal lobe in OI in healthy controls, and in normal aging the activity during OI decreases in these areas with age [11]-[16]. Cross-sectional and longitudinal volumetric studies have demonstrated a strong correlation between volume of the hippocampus and OI ability in AD [17]-[19]. Other non-CNS factors possibly affecting olfaction in AD include deposits of tau protein and beta-amyloid in the olfactory epithelium, and a reduced number of mitral cells and axonal loss in the olfactory tract [20]. OI performance has been shown to be associated with cognitive speed, verbal fluency, and memory in healthy controls and MCI/AD patients [5],[21]-[23].

There is currently little information regarding factors that differentiate patients with comparatively intact compared to impaired OI function in early AD, though OI has been found to predict conversion of MCI to AD [24],[25]. The main goal of this study was therefore to explore the relationship between the integrity of OI function, cognitive measures, and brain structure volume in healthy elderly individuals, compared to patients with aMCI or early AD. We also wished to examine whether differences in cognitive measures and brain structure volumes could be detected when patients were divided according to their OI ability.

## Methods

### Subjects and diagnostic procedure

In total 18 consecutive patients (eight males) recruited from the Memory Clinic, Department of Geriatrics, St. Olav Hospital (University Hospital of Trondheim) who agreed to participate, were included in this study. Patients were examined according to a standardized protocol for the Memory Clinic, following recommended international standards [26]. The diagnostic work-up with medical history was obtained from both patients and their caregivers. A clinical examination, including neuropsychology, cardiovascular status, neurological examination, and cerebral MRI at 3 T was performed which included the T1-weighted 3D volume from the Alzheimer Disease Neuroimaging (ADNI) MRI protocol. AD was diagnosed by an experienced geriatrician and assessed according to the criteria of the International Classification of Diseases (ICD-10) and the National Institute of Neurological and Communicative Disorders and Stroke and the Alzheimer’s Disease and Related Disorders Association (NINCDS-ADRDA) [27]. Patients fulfilling the accepted US National Institute on Aging-Alzheimer’s Association (NIA-AA) diagnostic criteria for aMCI were also included [28]. Of the 18 patients included, 12 were diagnosed with aMCI at baseline and six with early AD.

An additional 30 controls (16 males) were recruited by invitation through senior citizen centers, posters in the Trondheim area, alumni links, and personal networks. Patients and controls were excluded if they were not MRI-compatible, affected by some other serious somatic or psychiatric illness with an impact on activities of daily living, had nasal-sinus pathology, previous head trauma with loss of consciousness, brain infection, or had a viral infection at the time of the investigation.

All subsequent testing of participants was carried out at baseline. Relevant clinical data were gathered from patient records. Samples were taken for genomic DNA extraction from peripheral EDTA-blood and screened for *APOE* genotypes. Education level and smoking habits were recorded for each participant. In addition anterior rhinoscopy was performed in both patients and controls to check whether they had nasal polyps, tumor, or a pathological obstruction in the anterior nasal cavity. All individuals were checked for a history of olfactory, nasal and/or respiratory problems. A high resolution coronal T2-weighted scan of the sinuses and nasal cavity at 3 T (Siemens Trio scanner with a 12-channel Head Matrix Coil, Siemens AG, Erlangen, Germany) was obtained to identify any abnormalities in these structures not revealed by anterior rhinoscopy. To the best of our knowledge, no medications taken by patients or controls would have affected their olfactory performance.

All patients were assessed clinically 6–18 months (mean 9.9 months) later; 13 patients were then diagnosed with AD, and five still had aMCI. No tests were administered at follow-up as there was no ethical board approval for this. Since the writing of this manuscript, an additional patient with aMCI is known to have developed AD.

This study conformed to the Declaration of Helsinki. All participants received written information about the project and gave written consent. The study was approved by the Regional Committee of Medical Research Ethics for central Norway, and the Norwegian Data Inspectorate.

### Neuropsychological tests

The neuropsychological test battery included the Mini Mental Status Examination (MMSE), Trail Making Test A and B (TMT-A and TMT-B) [29], the Ten-Word Test (TWT) from the Consortium to Establish a Registry for Alzheimer’s Disease (CERAD), the Rey-Osterrieth Complex Figure Test (RCFT) [30], and a non-standardized Stereognosis Test in which the participants were presented with twelve different common objects (e.g. key, doll, pencil, clock and coin) placed in their hand without any visual cues (blindfolded).

### Psychophysical measurements

Olfactory measurements included The Brief Smell Identification Test (B-SIT, Sensonics Inc., Haddon Heights, USA), the Sniffin Sticks Identification Test (SSIT, Burghart Messetechnik GmbH, Wedel, Germany), and the Sniffin Sticks Discrimination Test (SSDT, Burghart Messetechnik GmbH, Wedel, Germany). SSDT was performed with only 16 of the patients. In B-SIT, a 12-item, four-choice, scratch-and-sniff identification test, the odorants are released from strips scratched with a special pencil. In SSIT the odor is released when removing the pen cap, with the pen positioned approximately two cm in front of both nostrils. Subjects were allowed to sniff SSIT-pens only once for three to four seconds. In both tests, the alternatives for the odorants were given orally twice by the experimenter, once before smelling the odor and again afterwards. The main difference in administration of the two tests was that in SSIT the test subjects could read the alternatives as well as hear them, whereas the alternatives in the B-SIT were written in English and not translated. The SSDT consists of 16 triplets, where two pens have the same smell, while one of the three pens contains a different odor. Participants were asked to identify the pen that had the different odor and were blindfolded because the pens were color-coded. Participants must choose one of the three pens of the triplet even when they do not perceive or recognize a different odor. In each olfactory test, correctly-identified odors received one point, giving a possible score range of 0–12 points for B-SIT, 0–16 for SSIT, and 0–16 for SSDT.

Subsequent comparison between the patients with aMCI and AD at baseline indicated no significant differences in performance on most tests, the only exceptions being weak significant differences with RCFT copying (p = 0.044), and TMT-A (p = 0.047). Taken together, as the groups were small and AD patients were in an early phase of disease (as assessed by MMSE: minimum score = 20, mean score = 24.8), both aMCI and AD patients were subsequently considered together as a single patient group for studying OI.

For further analysis of their OI abilities, this combined group of patients was divided according to performance on B-SIT and SSIT. For B-SIT the cut-off point was set to 6 such that patients scoring ≤ 6 (≤50%) were considered to have reduced OI ability (n = 6), and those scoring ≥7 (>50% score) were considered to have intact, or comparatively intact OI ability (n = 12). For SSIT, patients scoring ≤ 8 (≤50% score) were considered to have reduced OI ability, while those scoring ≥9 items correctly (>50% score) were considered to have intact, or comparatively intact OI ability.

The patient group was also split according to performance on delayed recall of the TWT (http://www.dia-online.no/tools/cerad%2010-ord%20fullstendig.doc), to indicate whether the results seen with the olfactory tests could be reproduced with a verbal memory test. Subgrouping patients below age 80 according to those scoring four or more (considered to indicate intact, or comparatively intact memory) or less than four (considered to indicate reduced memory), and at age 80 or over scoring 3 or more (intact, or comparatively intact memory) or less than three (reduced memory), resulted in a total group with at least comparatively intact memory of n = 6, and a group with reduced memory of n = 10 (n = 2 missing).

### Brain structural MRI volume

From all participants one T1-weighted 3D scan was obtained in the sagittal plane with repetition time (TR) = 2300 ms, echo time (TE) = 2.94 ms, field of view 256 mm, flip angle = 8, slice thickness = 1.00 mm, voxel size = 1.0*1.0*1.0 mm, slices per slab = 192, and base resolution = 256.

The T1 weighted volumes were analyzed using NeuroQuant (CorTechs Labs Inc., CA, USA) which performs automatic anatomical segmentation and volumetric measurement of brain structures (http://www.cortechs.net/index.php). Neuroquant is a fully automated segmentation FDA-cleared tool for clinical evaluation of hippocampal atrophy in mild cognitive impairment and Alzheimer’s disease [31]-[33]. The output from Neuroquant also includes other brain regions such as total hemispheric white matter, total cortical grey matter, lateral, third, fourth, and inferior lateral ventricle, cerebellum, amygdala, caudate, putamen, pallidum, thalamus and brainstem volumes. In this study brain regions related to olfactory processing were included in further analysis; total hemispheric white matter, cortical gray matter, thalamus, hippocampus, amygdala, and the total ventricular space. The volume of each structure was calculated as a percentage of the overall intracranial volume. The volume of structures such as the entorhinal cortex could not be determined by Neuroquant and is therefore not part of this study, although this region is considered central for both AD and OI [11],[34]. In this study volumes relative to ICV (% ICV) are used. The ICV-corrected volumes of the segmented structures from the left and right hemispheres were combined.

### Statistical analysis

Statistics were calculated using SPSS version 17 (SPSS Inc.). The B-SIT and SSIT tests were not normally distributed because they contain only unsigned integers. However, a Kolmogorov-Smirnov test on the whole material (patients and controls) indicated that the distribution of B-SIT and SSIT scores was not significantly different from normal (p > 0.1).

Psychophysical measurements, neuropsychological tests and volumetric measurements are reported as the mean ± SD. An independent samples *t*-testa and ANCOVA for correction of age, were used to test for significant differences between the patient and the control groups. Correlations between SSIT and B-SIT scores were assessed separately for the patient and control group. The chi-square test was used to compare occurrence of the *APOEε4* genotype, gender, smoking habits and education level in patients and controls. P-values less than 0.05 were considered significant.

Relationships between brain volumes or neuropsychological test results and the OI test scores subgrouped according to B-SIT or SSIT scores as “ > 50%” or “ ≤ 50%” were examined using Levene’s test for equality of variances with an independent *t*-test, plus a Bonferroni correction (3 comparisons), significance level adjusted to p < 0.017. Cohen’s *d* was used to measure the effect size of group differences between the “ > 50%” and “ ≤ 50%” OI groups. Group differences in brain volumes between the different groups were also assessed and corrected for age or TWT (delayed recall scores) in an ANCOVA, as appropriate. The kappa test was used to compare B-SIT and SSIT for discriminating patients from controls. Sensitivity and specificity were also calculated for B-SIT and SSIT. Linear regression analysis was carried out between hippocampal volume and the olfactory as well as the memory tests in the control and the patient group separately.

## Results

In this material, the combined group of patients with aMCI or AD was found to be significantly older than the control group, and had a much higher incidence of the *APOEε4* genotype (p = 0.001), but no significant differences were found in education level or smoking habits between the groups (Table 1). When patients were subdivided according to their performance on B-SIT (≥7 or ≤ 6 correct items), no significant differences were found in the demographic data, or any cognitive test, between patients in the two subgroups. When patients were divided according to their performance on SSIT (≥9 or ≤ 8 correct items), a significant difference in gender was found (Table 1). There was no significant difference in the distribution of the *APOEε4* allele between these subgroups.

**Table 1:** Demographic and test data for healthy elderly individuals (controls) and all patients with aMCI or early dementia in AD

			**Subgroups patients**			
			**B-SIT**		**SSIT**	
**Characteristics**	**Controls n = 30**	**Patients n = 18**	**>50% score n = 6**	**≤50% score n = 12**	**>50% score n = 10**	**≤50% score n = 6**
**Demographic data**						
Gender (female/male%)	46.7/53.3	55.6/44.4	66.7/33.3	50.0/50.0	70.0/30.0	16.7/83.3^+^
Age (years)	67.4 ± 7.6	74.6 ± 6.3**	76.0 ± 7.8	73.8 ± 5.7	76.5 ± 1.3	72.3 ± 5.1
Education (years)	17.1 ± 3.5	15.3 ± 2.5	15.3 ± 1.3	15.3 ± 3.0	14.8 ± 1.9	16.8 ± 3.4
Daily smokers (%)	3.3	5.6	0	8.3	0	0
*APOE*_Ɛ_4 genotype (% carriers 1–2 alleles)	20.8	73.3**	75.0	72.7	62.5	83.3
**Cognitive tests**						
MMSE (max. score 30)	28.7 ± 1.2	25.5 ± 2.5**	26.0 ± 1.7	25.3 ± 2.8	26.0 ± 1.6	25.7 ± 3.0
Ten-word test, total recall (max. score 30)	22.7 ± 3.6	12.5 ± 3.8**	13.2 ± 3.7	12.1 ± 3.9	12.1 ± 3.6	14.0 ± 4.2
Ten-words tests, delayed recall (max. 10)	8.1 ± 1.9	2.2 ± 1.7**	3.2 ± 1.8	1.6 ± 1.4	2.5 ± 1.9	1.8 ± 1.5
RCFT, figure copying	30.9 ± 3.0	26.0 ± 5.1**	24.6 ± 4.6	26.8 ± 5.4	23.9 ± 4.3	29.8 ± 4.4^+^
RCFT, immediate recall	17.9 ± 5.6	6.3 ± 4.4**	6.2 ± 6.1	6.4 ± 3.5	6.0 ± 4.6	6.8 ± 4.6
RCFT, delayed recall	17.7 ± 5.3	6.9 ± 4.2**	7.2 ± 6.8	6.7 ± 2.0	7.5 ± 4.8	5.9 ± 3.5
Stereognosis (max. score 12)	11.5 ± 0.8	10.2 ± 2.2**	10.2 ± 1.6	10.2 ± 2.6	10.6 ± 1.4	9.5 ± 3.2
Trail Making A (sec)	52.6 ± 20.1	64.7 ± 21.8	61.2 ± 15.9	64.2 ± 24.5	66.0 ± 21.4	66.7 ± 25.7
Trail Making-B (sec)	104.1 ± 37.5	134.0 ± 51.8*	141.0 ± 58.9	127.0 ± 48.3	148.3 ± 47.7	126.3 ± 56.2
**Phychophysical mesurements**						
B-SIT (12 items)	9.6 ± 2.0	6.6 ± 2.6**	9.8 ± 1.6	5.0 ± 0.7^##^	7.4 ± 2.6	5.7 ± 2.7
SSIT (16 items)	12.7 ± 2.4	9.4 ± 3.0**	11.3 ± 3.1	8.2 ± 2.3^#^	11.1 ± 2.1	6.5 ± 1.8^+^
SSDT (16 items)	9.2 ± 3.3	7.5 ± 3.0	7.8 ± 3.4	7.3 ± 3.0	7.4 ± 2.9	7.6 ± 3.5

Where appropriate, the mean ± SD is shown.

Significant differences using Student’s *t*-test for total group of patients compared to controls: *p < 0.05, **p < 0.005.

Significant differences using Student’s *t*-test for scoring >50% or ≤50% on B-SIT: ^#^p < 0.05, ^##^p < 0.005.

Significant differences using Student’s *t*-test for scoring >50% or ≤50% on SSIT: ^+^p < 0.05.

B-SIT; Brief Smell Identification Test, SSIT; Sniffin Sticks Identification Test, SSDT; Sniffin Sticks Discrimination Test, MMSE; Mini-Mental State Examination, RCFT; Rey-Osterrieth Complex Figure Test.

Patients have also been divided according to their odor identification (OI) ability.

### Performance on olfactory identification tests

**Figure 1:**
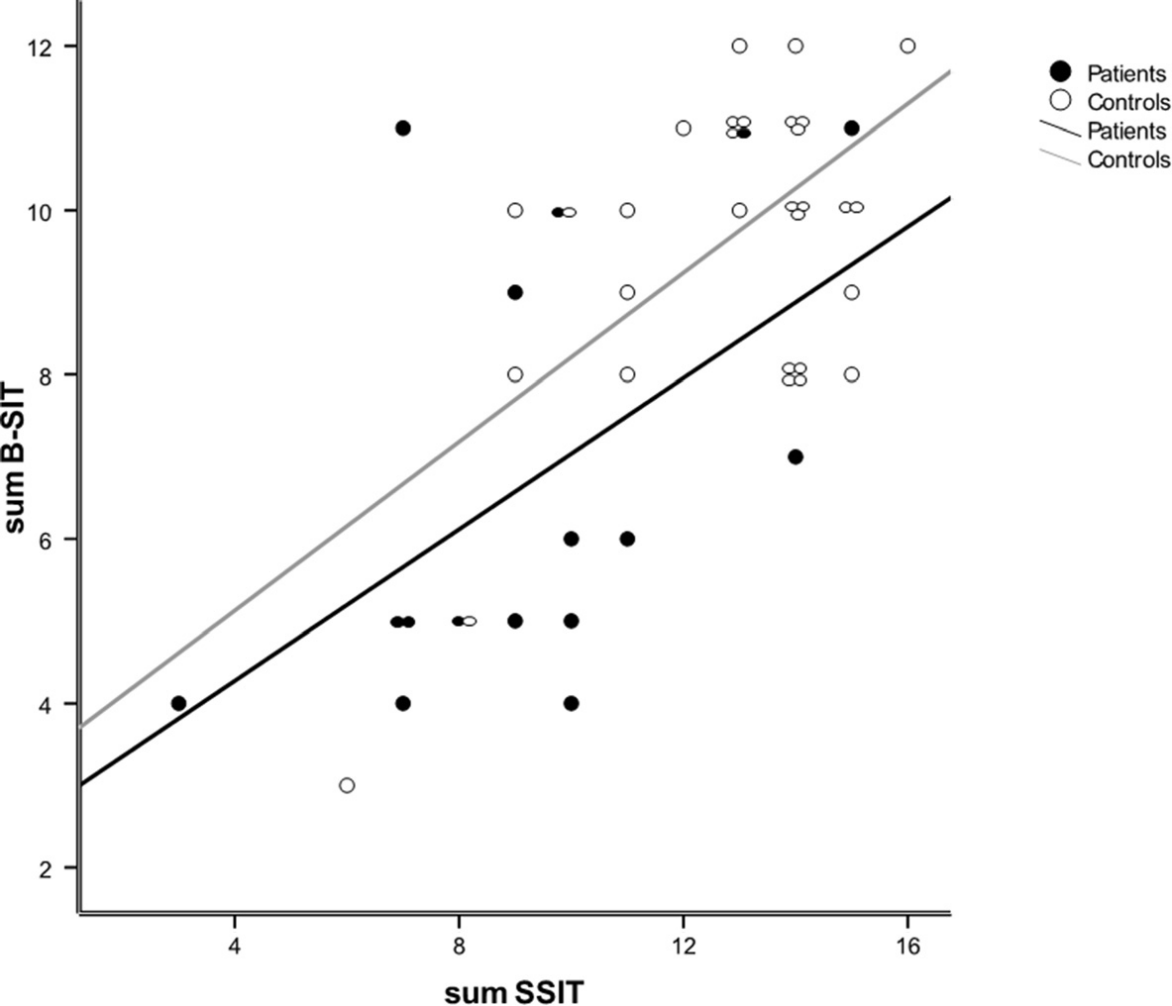
**Significant positive correlation between the Brief Smell Identification Test (B-SIT), or Sniffin Sticks Identification Test (SSIT) in both the control group (r = 0.606, p < 0.0005), and patient group (r = 0.514, p = 0.042)**.

Patients performed significantly worse than healthy controls on the two OI tests, but not on the odor discrimination test SSDT (Table 1). This result was unaltered when correcting for age. Figure 1 shows a positive correlation between B-SIT and SSIT in both the control (r = 0.606, p < 0.0005), and patient groups (r = 0.514, p = 0.042). Although all control individuals were considered cognitively intact at inclusion, it can be seen from the figure that two individuals, both men, had very low scores on both olfactory tests. However, they had MMSE scores of 27 and 29 respectively, and scored in the upper end of the TWT and RCFT. Nevertheless, B-SIT distinguished patients from controls when using a cut-off of ≥7/≤6 correct items [26], with a value of κ = 0.63, sensitivity 0.86 and specificity 0.82. Corresponding values for SSIT using a cut-off of ≥ 9/≤8 correct items were κ = 0.35, sensitivity 0.75 and specificity 0.74.

When patients were subdivided according to scores on B-SIT or SSIT, the number of patients scoring either > 50%, or ≤ 50% was not identical; more patients were considered to have comparatively intact OI by SSIT than B-SIT. However, all those considered to have comparatively intact OI by B-SIT were also considered so by the division according to SSIT. The five patients with aMCI who had not progressed to AD by 18 months were not concentrated into the > 50% group, but were divided 3 > 50% score: 2 ≤ 50% score with both tests. Neither OI score cut-off significantly distinguished results for SSDT (Table 1).

### Performance on cognitive tests

Patients performed significantly worse than healthy controls on all cognitive tests, with the exception of TMT-A (Table 1). The results were similar when correcting for age. The only difference found between the OI subgroups concerning the cognitive tests was significantly faster RCFT figure copying in the impaired OI group according to the division by SSIT (Table 1). There were many significant correlations between the cognitive and OI tests, but this aspect is well known [2],[4],[17],[35], and these results are not considered further in the present study.

### Odor identification and progression of aMCI to AD

The B-SIT score did not differentiate patients with aMCI from those with AD at baseline (p > 0.4), but prospectively differentiated those patients persisting with aMCI from those who had progressed to AD 6–18 months later (mean 9.9 months, *t*-test; p = 0.037). SSIT scores did not differentiate patients converting to AD. The respective percentages of patients judged to have reduced OI ability by B-SIT (≤50% score) for patients with aMCI who did not convert to AD during the follow-up period, patients with aMCI who converted to AD, and those with AD from baseline, were respectively 40%, 71% and 83%. Similar figures for SSIT were respectively 25%, 29% and 60%. Comparing patients with aMCI and AD at baseline did not show any significant differences in B-SIT scores, SSIT scores, or structural brain volumes.

### Structural MRI volumes in patients compared to controls, and to cognitive tests

Volumetric MRI measurement of brain structures demonstrated significantly smaller cortical gray matter in patients compared to controls, but no overall significant reduction in the volume of the hemispheric white matter between patients and controls even after correction for age. The most marked differences were found in hippocampus and amygdala, which were both significantly smaller compared to controls. The overall ventricular volume was significantly increased in patients compared to controls (Table 2). Regression analysis demonstrated that a reduction in the OI ability as measured by both B-SIT and SSIT was associated with smaller hippocampal volume in patients but not controls (Table 3). However, MMSE, TWT and RCFT (immediate and delayed recall) scores were not associated with hippocampal volume in patients. In the control group significant associations between hippocampal volume and MMSE (p = 0.014) and delayed recall of the Ten Word Test (p = 0.043) scores were found (Table 3).

**Table 2:** Volumetric measurements for several brain structures in patients (n = 18) and controls (n = 30)

**Brain areas**	**Control mean ± SD**	**Patients mean ± SD**
Hemispheric white matter	30.34 ± 1.90	29.37 ± 2.21
Cortical grey matter	29.46 ± 1.65	27.67 ± 1.84*
Hippocampus	0.49 ± 0.05	0.41 ± 0.04**
Amygdala	0.23 ± 0.02	0.20 ± 0.03**
Thalamus	1.20 ± 0.07	1.09 ± 0.07
Total ventricular volume	0.47 ± 0.15	0.74 ± 0.23**

Independent sample *t*-test (two-tailed): *p < 0.05 and **p < 0.005.

For bilateral structures volumes in the two hemispheres were combined, and all values are given as a percentage of total intracranial volume.

**Table 3:** Regression coefficients (linear regression analysis) between hippocampal volume in patients and controls, and odor identification and memory tests

**Tests**	**Hippocampal volume**	
	**Patients beta (p value)**	**Controls beta (p value)**
B-SIT	0.58 (0.012)*	0.10 (0.60)
SSIT	0.71 (0.002)*	0.22 (0.26)
MMSE	0.36 (0.18)	0.46 (0.014)*
Ten-word test, total recall	0.03 (0.91)	0.20 (0.30)
Ten-word test, delayed recall	0.21 (0.43)	0.38 (0.043)*
RCFT, figure copying	−0.53 (0.06)	0.03 (0.86)
RCFT, immediate recall	0.002 (0.99)	0.04 (0.85)
RCFT, delayed recall	0.15 (0.62)	0.12 (0.60)

Linear regression analysis: *p < 0.05

B-SIT; Brief Smell Identification Test, SSIT; Sniffin Sticks Identification Test, MMSE; Mini-Mental State Examination, RCFT; Rey-Osterrieth Complex Figure Test.

### Relationship between structural MRI volume and odor identification

Brain structure volume in patients were also compared according to the subdivisions of > 50% and ≤ 50% score on B-SIT or SSIT, and are shown in Table 4. The volume of hippocampus was significantly reduced in patients scoring ≤ 50% compared to those scoring > 50% regardless of whether OI was subdivided according to cut-offs for B-SIT or SSIT. Additionally, poor performance on both B-SIT and SSIT was associated with a significantly smaller hippocampal volume in the group defined as having ≤ 50% scores compared to that defined as having > 50% scores with a very large effect size (Cohen’s *d* = 1.3 for groups subdivided by B-SIT, and Cohen’s *d* = 1.9 for groups subdivided by SSIT). These differences remained significant after correction for performance on delayed recall of the TWT (B-SIT p = 0.011; SSIT p = 0.011). When the patient group was split according to performance on the TWT (delayed recall), no significant differences in hippocampal volume were found between the groups scoring > 50% compared to those scoring ≤ 50% on TWT.

**Table 4:** MRI volume of brain areas in patients with amnestic mild cognitive impairment or early Alzheimer’s disease, where patients have been subgrouped according to odor identification (OI) ability

	**B-SIT**		**SSIT**	
**Brain areas**	**Patients**		**Patients**	
	**>50% score n = 6 mean ± SD**	**≤50% score n = 12 mean ± SD**	**>50% score n = 10 mean ± SD**	**≤50% score n = 6 mean ± SD**
Hemispheric white matter	28.72 ± 2.49	29.40 ± 1.94	28.75 ± 1.96	30.49 ± 2.06
Cortical grey matter	27.77 ± 1.77	28.06 ± 1.25	28.15 ± 1.25	26.49 ± 2.46
Hippocampus	0.45 ± 0.02	0.41 ± 0.04^a^	0.44 ± 0.02	0.38 ± 0.04^a^
Amygdala	0.22 ± 0.04	0.18 ± 0.02	0.21 ± 0.03	0.17 ± 0.03^a^
Thalamus	1.10 ± 0.07	1.10 ± 0.08	1.13 ± 0.07	1.20 ± 0.56
Total venticular volume	0.86 ± 0.16	0.65 ± 0.22	0.81 ± 0.24	0.72 ± 0.18

^a^Significant difference between patients subgrouped as having > 50% or ≤ 50% score according to either the Brief Smell Identification Test (B-SIT), or Sniffin Sticks Identification Test (SSIT), p < 0.017.

Volumes of both cortical gray matter and amygdala were significantly smaller in the patient subgroup scoring ≤ 50%, again according to the cut-off for both B-SIT and SSIT, compared to controls (Table 2). However, amygdala volume was significantly less in the patient subgroup scoring ≤ 50% compared to those scoring > 50% only according to the cut-off for SSIT, again with a large effect size (Cohen’s *d* = 1.3 for groups subdivided by SSIT). A smaller amygdale size was also observed with B-SIT, but did not reach the level of significance.

## Discussion

The number of patients included in the present study was low, with a high mean age. It was both difficult to recruit patients from the geriatric memory clinic, and difficult for those volunteering to fulfill all inclusion criteria and perform all the tests. However, the most important result in the present study is that impaired OI performance, as measured by two separate olfactory tests, B-SIT and SSIT, is related to differences in the volume of several brain structures, most particularly the hippocampus. Notably, this difference in volumes was found within the patient group when subdivided according to performance on the two OI tests (divided according to those with comparatively intact OI (>50% score) and those with reduced OI (≤50% score on B-SIT and SSIT), as well as between a group consisting of patients with aMCI or AD, and healthy control individuals. Validated cut-offs for olfactory tests have been established [8],[24], but in the present small study the main aim was to compare patients on the basis of changes in the volume of brain areas with their OI abilities, as well as changes in memory. We also wished to see if the results were similar in two separate OI tests, which required establishing a cut-off for each that should bear a relationship to each other, where each test would reflect a similar change in OI ability (individuals with comparatively intact OI performance, as opposed those with little or no OI ability). Although this division is arbitrary, those with 50% scores or less will probably have a compromised OI, and it is unlikely that patients will achieve over 50% on such tests purely by chance (achieving 7 correct answers on B-SIT by chance is p = 0.011, and achieving 9 correct answers on SSIT by chance is p = 0.006). That the patients in the subgroup scoring ≤ 50% had smaller hippocampal volume than the subgroup scoring >50% on the olfactory tests, suggests that the former subgroup had greater atrophy in this brain area. Despite this being a study with small groups, the effect size was calculated to be from high to very high. It is of course possible that some of the controls, such as the two with the very low OI scores, could in time develop AD or another neurodegenerative condition. However, pathological tendencies in the control group would probably have reduced the difference with patient group rather than accentuating it.

The cognitive tests (with the exception of TMT-A), odor tests (except SSDT), and volumetric MRI (with the exception of cortical white matter and thalamus), were all capable of clearly distinguishing the patient group from healthy elderly controls, as expected [2],[6],[17]. These results from B-SIT and SSIT support previous studies that the group average of OI scores is lower in patients with AD and MCI compared to healthy controls [1],[6],[17]. Moreover, B-SIT and SSIT correlated well with each other in both groups.

Within the patient group, neuropsychological tests applied did not distinguish between the OI subgroups. The cognitive tests in general did not tend to show an association with hippocampal volume, though MMSE and the delayed recall part of the TWT were found to correlate significantly among controls. MMSE scores for controls were all ranged from the maximum 30 points to a minimum of 27, whereas patient scores ranged from 28 to a minimum of 20 points. Apart from the small size of the patient group, other factors can play a role in these tests, even among cognitively intact elderly individuals. In addition to age, these may include aspects such as the level of education and other psychological conditions. Such aspects may contribute to a spread of memory ability across a control group, but result in a different association in connection with a patient group with a progressive dementia, in addition to other environmental and age-related aspects.

It could be argued that as the patient group was older than the controls, differences in cognition could be accentuated, since both neuropsychological and odor tests have been shown to correlate with age [36],[37]. However, the statistical differences between the control and patient groups remained significant after correction for age. Furthermore, there was no significant difference in age between the patient subgroups. Thus, parameters other than age most likely contributed to the differences in OI test scores between the subgroups. It seems unlikely that smoking could have affected the results as only one smoker was present in the control group, and one in the patient group. This patient did show reduced OI ability on B-SIT (and did not carry out SSIT), but was a patient with a diagnosis of AD at baseline, and as a group, these patients had low B-SIT scores.

In the patient group, the negative beta value found for the figure-copying part of the RCFT and hippocampal volume, though only a strong trend, suggests that patients with smaller hippocampal volume copy the figure faster than those with a larger volume. Copying of course does not require memory. It is interesting that this part of the RCFT was performed better by the patient group scoring ≤50% on SSIT. A larger test group would be needed to confirm whether this result is correct or a chance finding.

Apart from these aspects, the cognitive tests in general did not tend to show an association with hippocampal volume. Although it is important to treat results from small studies with care, the most consistent results here were obtained with B-SIT and SSIT.

The results showed that the two OI tests subdivided the patient group differently, the cut-off for SSIT defining only six patients with scores of ≤ 50%, whereas 12 patients scored 50% or less with B-SIT. A possibility for this may well have been because the tests themselves involved slightly different approaches to measuring olfaction: with B-SIT, information concerning the odor alternatives was only given orally, whereas with SSIT, alternatives were available both orally and visually (they could be read). The additional visual cue in SSIT was perhaps enough to improve the performance of certain patients on this test. Of the six patients who scored inconsistently on the two tests, three were only borderline differences. Indeed, although more patients were considered to have only slight loss or normal OI according to the cut-off for SSIT, all those considered so by B-SIT were among them. This may also explain why both tests tended to show similarities in the volumetric data despite the difference in OI group-assignment.

There was no clear connection found between the clinical diagnosis, and the olfactory and volumetric data. It might have been expected that patients maintaining a diagnosis of aMCI throughout the study would have better OI according to the olfactory tests, while those with AD, as well as those progressing to AD, would more often display reduced OI. Some evidence for such a relationship between B-SIT and disease progression was evident, as shown by the prospective difference between mean B-SIT scores between patients with persisting aMCI compared to those with AD at follow-up, and the increases in percentage of patients showing a reduction in OI ability between those maintaining aMCI over the follow-up period, those converting to AD, and those with AD at baseline. However, our results overall further support observations that loss of OI ability tends to occur early in AD, although this is not absolute [1].

That so many patients converted from aMCI to AD in a short space of time in this study perhaps helps explain why there were so few statistical differences between patients with aMCI compared to AD on the cognitive tests. A higher conversion rate should be expected when only patients with aMCI are included, rather than a mixed group including non-amnestic MCI. Another cohort followed by us longitudinally over 2 years, and younger on average by 10 years than patients in the study reported here, also shows a high percentage of conversion (49%) within this period (unpublished results). The present cohort was older, so the period between aMCI being diagnosed and onset of dementia may well be accelerated as cognitive reserve declines with increasing age. All the patients in this study were in an early stage of disease, so it is not surprising that neither marked differences in OI ability, nor reductions in brain structural volume according to diagnosis were found. Clinically, it is not easy to clearly differentiate aMCI from early AD, and recent proposals for harmonizing new diagnostic criteria will attempt to simplify and standardize terminology in AD, whether prodromal or in the dementia phase [38]. Simple tests that help determine early changes are necessary for identifying individuals most at risk, and in our study, olfaction rather than memory, seemed to relate better to the size of hippocampus.

## Conclusions

The results of this study suggest that competence in olfactory identification rather than memory is associated with the volume of several brain structures, particularly hippocampus in aMCI and AD. This result was found regardless of whether the B-SIT or SSIT olfactory test was employed, and not only distinguished patients with aMCI and early AD from healthy control individuals, but also suggested that patients with greater olfactory impairment have increased brain atrophy. These results require confirmation in a larger patient population.

## Competing interests

The authors declare that they have no competing interests.

## Authors’ contributions

AKH, KE, OS and GK designed the study; IS, OS, AKL and PS recruited patients; GK carried out the psychophysical tests, GK, KS and HAS carried out the volumetric MR; KS and HAS carried out the neuropsychological testing; AKH and GK calculated the MRI data; GK, LRW, KE and AKH carried out and controlled the statistics; GK, LRW, IS, KE, OS and AKH drafted the manuscript. All authors read and approved the final manuscript.
